# Hygiene: microbial strategies to reduce pathogens and drug resistance in clinical settings

**DOI:** 10.1111/1751-7915.12755

**Published:** 2017-07-05

**Authors:** Elisabetta Caselli

**Affiliations:** ^1^ CIAS Section of Microbiology Department of Medical Sciences University of Ferrara via L. Borsari 46 44121 Ferrara Italy

## Abstract

Healthcare‐associated infections (HAIs) are a global concern, affecting all western hospitals, and profoundly impairing the clinical outcome of up to 15% of all hospitalized patients. Persistent microbial contamination of hospital surfaces has been suggested to contribute to HAIs onset, representing a reservoir for hospital pathogens. On the other hand, conventional chemicals‐based sanitation do not prevent recontamination and can select drug‐resistant strains, resulting in over 50% of surfaces persistently contaminated. There is therefore an urgent need for alternative sustainable and effective ways to control pathogens contamination and transmission. Toward this goal, we recently reported that a probiotic‐based sanitation can stably decrease surface pathogens up to 90% more than conventional disinfectants, without selecting resistant species. This paper summarizes some of our most significant results.

Trying to ensure a healthy environment during hospitalization represents a vital sustainable development goal of the recent years, in the attempt to face up to the global concern of hospital acquired infections.

Such infections affect up to 15% of all hospitalized patients in high‐income countries (Allegranzi *et al*., 2011; Cookson *et al*., 2013; Suetens *et al*., 2013) and represent the fourth largest killer in the USA (Pharmaceutical, 2015).

Just because of their illnesses, hospital inpatients are predisposed to both contracting and spreading infections, and hospital surfaces can represent the reservoir for the associated pathogens.

In fact, the contribution of contaminated hospital surfaces is increasingly recognized as an important factor in the acquisition of infections associated with hospitalization (healthcare‐associated infections, HAIs).

So far, the control of surface contamination has been addressed almost exclusively by use of chemical compounds, which are accompanied by a non‐negligible environmental impact (Kummerer, 2001), and show important limitations.

First, although disinfectants can be effective in the immediate abatement of surface pathogens, they result ineffective in preventing recontamination phenomena, occurring as fast as 30 min, which are ultimately responsible for pathogens persistence. In agreement with this observation, several studies have shown that at least half of hospital surfaces are inadequately sanitized by use of chemical germicides (Carling *et al*., 2008; Goodman *et al*., 2008). Many pathogens are in fact persisting for long periods on high, medium or low touch nosocomial surfaces (Hota, 2004; Kramer *et al*., 2006; Boyce *et al*., 2011; Huslage *et al*., 2013). Staphylococci, including methicillin resistant *Staphylococcus aureus* (MRSA), vancomycin‐resistant enterococci (VRE), *Pseudomonas* spp., *Acinetobacter* spp. and even viruses (i.e. norovirus), are retaining their infectivity for days to months on dry inanimate surfaces (Weber and Rutala, 1997; Kramer *et al*., 2006; Boyce, 2007; Weber *et al*., 2010). *Clostridium difficile* spores are indeed surviving for several months on environmental surfaces and contaminate about 75% of rooms hosting infected patients (Weber *et al*., 2010).

Second, disinfectants can select resistant microbial strains against the disinfectant itself (Bock *et al*., 2016), and also, more importantly, against antibiotics, as recently reported for chlorhexidine induction of resistance against Colistin (Wand *et al*., 2017), an antibiotic considered till 2016 as a last‐resort drug for treatment of infections sustained by multidrug‐resistant (MDR) Gram‐negative bacteria.

The potential induction of antibiotic resistance represents a highly undesirable side‐effect of chemical cleaning, as MDR pathogens have been constantly and rapidly growing in the recent decades and a high proportion of HAIs is caused by MDR bacteria (Caini *et al*., 2013; Cornejo‐Juarez *et al*., 2015), threatening the outcome of an ever‐increasing range of infections.

Thus, taken together, these data suggest that chemical sanitation, although well‐intentioned, cannot guarantee a true healthy environment for patients, as it appears unable to maintain the environment safe and might even increase bacterial drug resistance.

Alternative methods to chemical disinfectants have been proposed to control surface contamination, including the use of ‘self‐disinfecting’ surfaces based on the use of heavy metals (silver, copper), germicide impregnated materials or light‐activated antimicrobial coatings (Carling and Bartley, 2010; Dancer, 2011; Davies *et al*., 2011; Rutala and Weber, 2011, 2013; Otter *et al*., 2013). However, these methods result very expensive and not suitable for all types of surfaces and settings, including those in low‐income countries.

Based on these observations, there is an urgent need for sustainable effective alternatives to the use of conventional germicides.

In the attempt to minimize the infectious risk for hospitalized patients, and to avoid increasing of drug resistance and environmental impact, the sanitation of hospital surfaces has been recently rethought, trying to manage the ‘health’ of the hospital environment as the health of the human body.

This approach, inspired by the Microbiome Project data, considers that, rather than eradicating all pathogens, replacing pathogens by beneficial microbes might be more effective in decreasing infections (Al‐Ghalith and Knights, 2015; Pettigrew *et al*., 2016).

It is in fact generally accepted that beneficial microbes are important for our health and that their use can be effective in the prevention and treatment of infectious diseases (Koenigsknecht and Young, 2013). Among the microorganisms potentially useful towards this aim, probiotics appear particularly interesting, as they are defined as beneficial microbes for our health, capable to ‘fill the void’, disadvantaging the colonization by pathogens (WHO, 2001; Hill *et al*., 2014). Notably, probiotics have been shown effective in reducing the occurrence of different nosocomial infections, including diarrhoea, necrotizing enterocolitis (Giamarellos‐Bourboulis *et al*., 2009), upper respiratory infections (Banupriya *et al*., 2015) and infections in surgical patients (Rayes *et al*., 2002, 2012; Sommacal *et al*., 2015).

On that basis, for several years we have been working on a sanitation approach based on the addition of spores of probiotics belonging to the *Bacillus* genus to eco‐sustainable detergents. In this study, some of our most significant results are reviewed.

Bacteria of the *Bacillus* genus are apathogenic (except for two well‐recognizable species) (EFSA, 2010), ubiquitous (they are present in soil, water, vegetables, as in human gut) and have a long history of safe use in humans. Furthermore, spore former probiotics are particularly suitable for addition to detergents, as spores maintain their viability in the concentrated cleanser, originating the vegetative bacteria when diluted in water and seeded on surfaces.

The system, named Probiotic Cleaning Hygiene System (PCHS) and including three *Bacillus* species (*B. subtilis*,* B. pumilus*,* B. megaterium*), was tested in ten different hospitals, in Italy and Belgium, both by simultaneous comparison of wards treated by PCHS and conventional disinfectants, and by sequential pre‐ to post‐comparison of surface bioburden in the same wards treated sequentially with both systems. The results, collected in over 4 years, showed that PCHS stably decreased the presence of pathogens on treated surfaces, about 90% more than conventional cleansers (Vandini *et al*., 2014; Caselli *et al*., 2016a) (Fig. 1A). This effect was associated with germination of probiotic *Bacillus* spores, followed by an actual replacement of pathogens by PCHS *Bacillus*, reaching about 70% of the total surface microbiota after 1 month of application (Caselli *et al*., 2016a). Importantly, PCHS did not select any drug‐resistant strain, but rather it induced a general decrease in the whole antibiotic resistance genes of the residual microbiota, compared to the original pre‐existing one (Caselli *et al*., 2016a) (Fig. 1B).

**Figure 1:**
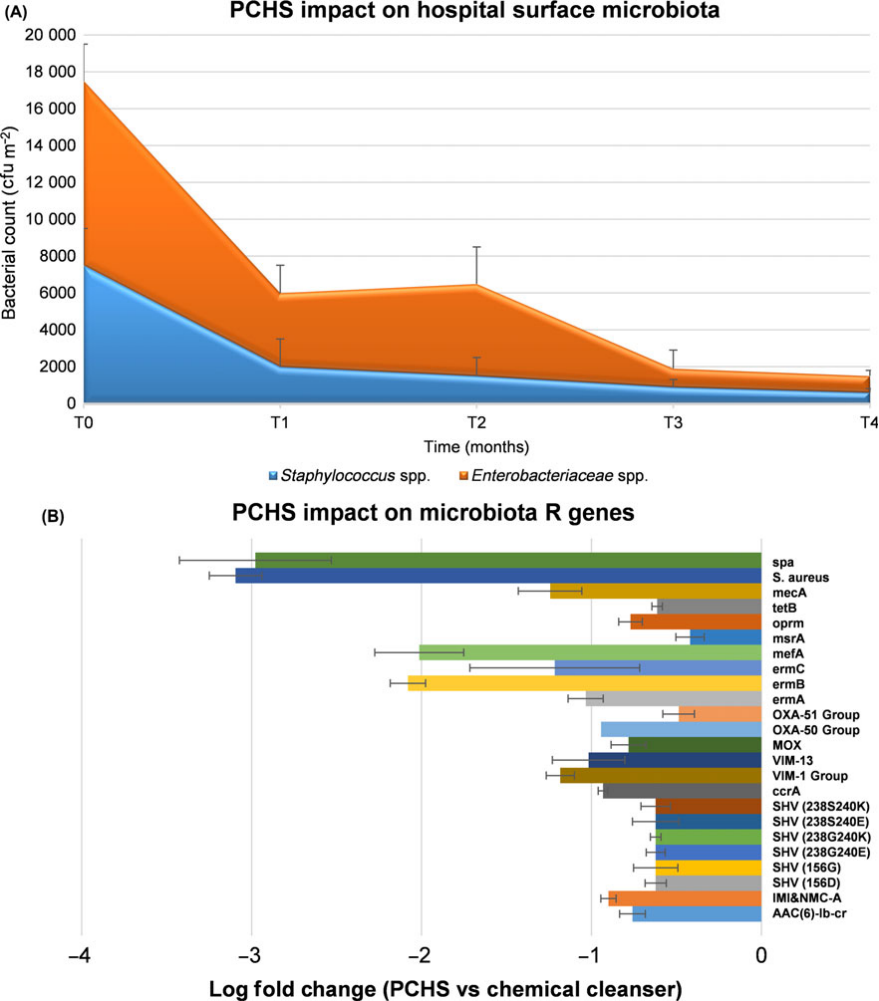
Impact of the probiotic‐based microbial cleaning on microbiota contaminating hospital surfaces. A. PCHS effect on Gram‐positive (*Staphylococcus* spp.) and Gram‐negative (*Enterobacteriaceae* spp.) pathogens amounts on treated surfaces after 1, 2, 3, 4 months of PCHS continuous sanitation (T0 values are those obtained with chemical sanitation); results are expressed as median CFU counts per m^2^. B. PCHS effect on the R genes of the whole residual microbiota (resistome) after 1–4 months of PCHS sanitation; results were obtained by PCR microarray and are expressed as of log fold change in R genes compared to the values detected at T0; mean values of the 4 months ± SD are reported.

In addition, probiotic *Bacilli* resulted genetically very stable, as they did not acquire any new resistance gene over a period of 4 years of continuous use, despite the continuous contact with surface pathogenic and drug‐resistant neighbours (Caselli *et al*., 2016a).

Last, due to the enzymatic activity of probiotic *Bacilli*, producing esterases, lipases, glucosidases and phosphatases (unpublished personal observations), their addition to cleansers allowed to abate the need for high concentrations of chemical detergent compounds in the cleanser itself, resulting in eco‐sustainable formulations with low environmental impact and low cost.

However, as one of the major obstacles still limiting the use of probiotics for hospital sanitation regards their theoretical infectious risk, we wanted to address this point, as a few anecdotic cases of adverse events had been reported in the past (Logan, 1988), and there is a lack of systematic studies on probiotics safety (Doron and Snydman, 2015).

Thus, we implemented a microbiological surveillance for *Bacillus* in the healthcare structures continuously using PCHS up to 4 years, analysing for *Bacillus* presence all the clinical samples derived from hospitalized patients. Over 32 000 clinical specimens from subjects with and without HAIs were analysed, and no positive samples were found (Caselli *et al*., 2016b), even using highly sensitive molecular techniques (Caselli *et al*., 2016a,b), suggesting that probiotic *Bacilli* do not represent an infectious risk even in the particularly susceptible hospitalized patient.

Taken together, these data show that a sanitation system based on the principle of competitive antagonism between good and bad bacteria can be highly effective in reducing and maintaining stably low the level of dangerous pathogens in hospital environment. In addition, the absence of selection of resistant species can be of further help in reducing the risk of hazardous and hard‐to‐treat infections in hospitalized subjects. Last, the costs of this system are even lower compared to those of chemical disinfectants, thus rendering it suitable for a widespread application without additional costs for the healthcare structures.

At the moment, the main limitation of probiotic‐based sanitation is represented by the time needed to obtain a stable microbial balance on surfaces (2–4 weeks), that makes it ideal for a prophylactic use, rather than for instantaneous decontamination. This feature can be substantially ameliorated and will deserve future studies. Also, it will be necessary to verify whether the decrease in surface drug‐resistant pathogens corresponds to a really diminished number of developed HAIs. To this aim, we are currently performing a 18‐month multicenter study in seven Italian hospitals, involving about 19 000 patients hosted in PCHS‐treated structures (Caselli *et al*., 2016c), as the decrease of infections in hospitalized patients is the final goal of these researches.

We are convinced that, when using microbiological agents, a constant monitoring by microbiological and molecular analyses is mandatory, as it can guarantee both safety and effectiveness optimization of the microbial procedure.

However, based on the data collected so far, we feel that probiotic strategies may significantly advance efforts towards reducing infections in hospitals and likely in other environments (home care, breeding farms, etc.), thus deeply improving health sustainability.

## Conflict of interest

The author has no conflict of interest to declare.

## References

[mbt212755-bib-0001] Al‐Ghalith, G.A. , and Knights, D. (2015) Bygiene: the New Paradigm of Bidirectional Hygiene. Yale J Biol Med 88: 359–365. 26604859 PMC4654184

[mbt212755-bib-0002] Allegranzi, B. , Bagheri Nejad, S. , Combescure, C. , Graafmans, W. , Attar, H. , Donaldson, L. , and Pittet, D. (2011) Burden of endemic health‐care‐associated infection in developing countries: systematic review and meta‐analysis. Lancet 377: 228–241. 21146207 10.1016/S0140-6736(10)61458-4

[mbt212755-bib-0003] Banupriya, B. , Biswal, N. , Srinivasaraghavan, R. , Narayanan, P. , and Mandal, J. (2015) Probiotic prophylaxis to prevent ventilator associated pneumonia (VAP) in children on mechanical ventilation: an open‐label randomized controlled trial. Intensive Care Med 41: 677–685. 25708419 10.1007/s00134-015-3694-4

[mbt212755-bib-0004] Bock, L.J. , Wand, M.E. , and Sutton, J.M. (2016) Varying activity of chlorhexidine‐based disinfectants against *Klebsiella pneumoniae* clinical isolates and adapted strains. J Hosp Infect 93: 42–48. 26899354 10.1016/j.jhin.2015.12.019

[mbt212755-bib-0005] Boyce, J.M. (2007) Environmental contamination makes an important contribution to hospital infection. J Hosp Infect 65(Suppl 2): 50–54. 17540242 10.1016/S0195-6701(07)60015-2

[mbt212755-bib-0006] Boyce, J.M. , Havill, N.L. , Havill, H.L. , Mangione, E. , Dumigan, D.G. , and Moore, B.A. (2011) Comparison of fluorescent marker systems with 2 quantitative methods of assessing terminal cleaning practices. Infect Control Hosp Epidemiol 32: 1187–1193. 22080657 10.1086/662626

[mbt212755-bib-0007] Caini, S. , Hajdu, A. , Kurcz, A. and Borocz, K. (2013) Hospital‐acquired infections due to multidrug‐resistant organisms in Hungary, 2005‐2010. Euro Surveill 18: 1–9. 23324427

[mbt212755-bib-0008] Carling, P.C. , and Bartley, J.M. (2010) Evaluating hygienic cleaning in health care settings: what you do not know can harm your patients. Am J Infect Control 38: S41–S50. 20569855 10.1016/j.ajic.2010.03.004

[mbt212755-bib-0009] Carling, P.C. , Parry, M.F. , and Von Beheren, S.M. (2008) Identifying opportunities to enhance environmental cleaning in 23 acute care hospitals. Infect Control Hosp Epidemiol 29: 1–7. 18171180 10.1086/524329

[mbt212755-bib-0010] Caselli, E. , D'Accolti, M. , Vandini, A. , Lanzoni, L. , Camerada, M.T. , Coccagna, M. , *et al* (2016a) Impact of a probiotic‐based cleaning intervention on the microbiota ecosystem of the hospital surfaces: focus on the resistome remodulation. PLoS ONE 11: e0148857. 26886448 10.1371/journal.pone.0148857 PMC4757022

[mbt212755-bib-0011] Caselli, E. , Antonioli, P. , and Mazzacane, S. (2016b) Safety of probiotics used for hospital environmental sanitation. J Hosp Infect 94: 193–194. 27436618 10.1016/j.jhin.2016.06.021

[mbt212755-bib-0012] Caselli, E. , Berloco, F. , Tognon, L. , Villone, G. , La Fauci, V. , Nola, S. , *et al* (2016c) Influence of sanitizing methods on healthcare‐associated infections onset: a multicentre, randomized, controlled pre‐post interventional study. J Clin Trials 6: 6.

[mbt212755-bib-0013] Cookson, B. , Mackenzie, D. , Kafatos, G. , Jans, B. , Latour, K. , Moro, M.L. , *et al* (2013) Development and assessment of national performance indicators for infection prevention and control and antimicrobial stewardship in European long‐term care facilities. J Hosp Infect 85: 45–53. 23932737 10.1016/j.jhin.2013.04.019

[mbt212755-bib-0014] Cornejo‐Juarez, P. , Vilar‐Compte, D. , Perez‐Jimenez, C. , Namendys‐Silva, S.A. , Sandoval‐Hernandez, S. , and Volkow‐Fernandez, P. (2015) The impact of hospital‐acquired infections with multidrug‐resistant bacteria in an oncology intensive care unit. Int J Infect Dis 31: 31–34. 25528484 10.1016/j.ijid.2014.12.022

[mbt212755-bib-0015] Dancer, S.J. (2011) Hospital cleaning in the 21st century. Eur J Clin Microbiol Infect Dis 30: 1473–1481. 21499954 10.1007/s10096-011-1250-x

[mbt212755-bib-0016] Davies, A. , Pottage, T. , Bennett, A. , and Walker, J. (2011) Gaseous and air decontamination technologies for *Clostridium difficile* in the healthcare environment. J Hosp Infect 77: 199–203. 21130521 10.1016/j.jhin.2010.08.012

[mbt212755-bib-0017] Doron, S. , and Snydman, D.R. (2015) Risk and safety of probiotics. Clin Infect Dis 60(Suppl 2): S129–S134. 25922398 10.1093/cid/civ085 PMC4490230

[mbt212755-bib-0018] EFSA (2010) Panel on Biological Hazards (BIOHAZ). Scientific opinion on the maintenance of the list of QPS microorganisms intentionally added to food or feed (2010 update). EFSA J 8: 1–89.

[mbt212755-bib-0019] Giamarellos‐Bourboulis, E.J. , Bengmark, S. , Kanellakopoulou, K. , and Kotzampassi, K. (2009) Pro‐ and synbiotics to control inflammation and infection in patients with multiple injuries. J Trauma 67: 815–821. 19820590 10.1097/TA.0b013e31819d979e

[mbt212755-bib-0020] Goodman, E.R. , Platt, R. , Bass, R. , Onderdonk, A.B. , Yokoe, D.S. , and Huang, S.S. (2008) Impact of an environmental cleaning intervention on the presence of methicillin‐resistant Staphylococcus aureus and vancomycin‐resistant enterococci on surfaces in intensive care unit rooms. Infect Control Hosp Epidemiol 29: 593–599. 18624666 10.1086/588566 PMC2670228

[mbt212755-bib-0021] Hill, C. , Guarner, F. , Reid, G. , Gibson, G.R. , Merenstein, D.J. , Pot, B. , *et al* (2014) Expert consensus document. The international scientific association for probiotics and prebiotics consensus statement on the scope and appropriate use of the term probiotic. Nat Rev Gastroenterol Hepatol 11: 506–514. 24912386 10.1038/nrgastro.2014.66

[mbt212755-bib-0022] Hota, B. (2004) Contamination, disinfection, and cross‐colonization: are hospital surfaces reservoirs for nosocomial infection? Clin Infect Dis 39: 1182–1189. 15486843 10.1086/424667 PMC7107941

[mbt212755-bib-0023] Huslage, K. , Rutala, W.A. , Gergen, M.F. , Sickbert‐Bennett, E.E. , and Weber, D.J. (2013) Microbial assessment of high‐, medium‐, and low‐touch hospital room surfaces. Infect Control Hosp Epidemiol 34: 211–212. 23295570 10.1086/669092

[mbt212755-bib-0024] Koenigsknecht, M.J. , and Young, V.B. (2013) Faecal microbiota transplantation for the treatment of recurrent *Clostridium difficile* infection: current promise and future needs. Curr Opin Gastroenterol 29: 628–632. 24100717 10.1097/MOG.0b013e328365d326 PMC4127992

[mbt212755-bib-0025] Kramer, A. , Schwebke, I. , and Kampf, G. (2006) How long do nosocomial pathogens persist on inanimate surfaces? A systematic review. BMC Infect Dis 6: 130. 16914034 10.1186/1471-2334-6-130 PMC1564025

[mbt212755-bib-0026] Kummerer, K. (2001) Drugs in the environment: emission of drugs, diagnostic aids and disinfectants into wastewater by hospitals in relation to other sources–a review. Chemosphere 45: 957–969. 11695619 10.1016/s0045-6535(01)00144-8

[mbt212755-bib-0027] Logan, N.A. (1988) Bacillus species of medical and veterinary importance. J Med Microbiol 25: 157–165. 3279213 10.1099/00222615-25-3-157

[mbt212755-bib-0028] Otter, J.A. , Yezli, S. , Perl, T.M. , Barbut, F. , and French, G.L. (2013) The role of ‘no‐touch’ automated room disinfection systems in infection prevention and control. J Hosp Infect 83: 1–13. 23195691 10.1016/j.jhin.2012.10.002

[mbt212755-bib-0029] Pettigrew, M.M. , Johnson, J.K. , and Harris, A.D. (2016) The human microbiota: novel targets for hospital‐acquired infections and antibiotic resistance. Ann Epidemiol 26: 342–347. 26994507 10.1016/j.annepidem.2016.02.007 PMC4892961

[mbt212755-bib-0030] Pharmaceutical, I.M. (2015) RID Study: infection caught in the US hospitals is the 4th biggest killer.

[mbt212755-bib-0031] Rayes, N. , Seehofer, D. , Hansen, S. , Boucsein, K. , Muller, A.R. , Serke, S. , *et al* (2002) Early enteral supply of lactobacillus and fiber versus selective bowel decontamination: a controlled trial in liver transplant recipients. Transplantation 74: 123–127. 12134110 10.1097/00007890-200207150-00021

[mbt212755-bib-0032] Rayes, N. , Pilarski, T. , Stockmann, M. , Bengmark, S. , Neuhaus, P. , and Seehofer, D. (2012) Effect of pre‐ and probiotics on liver regeneration after resection: a randomised, double‐blind pilot study. Benef Microbes 3: 237–244. 22968413 10.3920/BM2012.0006

[mbt212755-bib-0033] Rutala, W.A. , and Weber, D.J. (2011) Are room decontamination units needed to prevent transmission of environmental pathogens? Infect Control Hosp Epidemiol 32: 743–747. 21768756 10.1086/661226

[mbt212755-bib-0034] Rutala, W.A. , and Weber, D.J. (2013) Disinfectants used for environmental disinfection and new room decontamination technology. Am J Infect Control 41: S36–S41. 23622746 10.1016/j.ajic.2012.11.006

[mbt212755-bib-0035] Sommacal, H.M. , Bersch, V.P. , Vitola, S.P. , and Osvaldt, A.B. (2015) Perioperative synbiotics decrease postoperative complications in periampullary neoplasms: a randomized, double‐blind clinical trial. Nutr Cancer 67: 457–462. 25803626 10.1080/01635581.2015.1004734

[mbt212755-bib-0036] Suetens, C. , Hopkins, S. , Kolman, J. , and Diaz Högberg, L. (2013) Point Prevalence Survey of Healthcare Associated Infections and Antimicrobial Use in European Acute Care Hospitals. Stockholm, Sweden: European Centre for Disease Prevention and Control.

[mbt212755-bib-0037] Vandini, A. , Temmerman, R. , Frabetti, A. , Caselli, E. , Antonioli, P. , Balboni, P.G. , *et al* (2014) Hard surface biocontrol in hospitals using microbial‐based cleaning products. PLoS ONE 9: e108598. 25259528 10.1371/journal.pone.0108598 PMC4178175

[mbt212755-bib-0038] Wand, M.E. , Bock, L.J. , Bonney, L.C. and Sutton, J.M. (2017) Mechanisms of increased resistance to chlorhexidine and cross‐resistance to colistin following exposure of *Klebsiella pneumoniae* clinical isolates to chlorhexidine. Antimicrob Agents Chemother 61: e01162–16. 27799211 10.1128/AAC.01162-16 PMC5192135

[mbt212755-bib-0039] Weber, D.J. , and Rutala, W.A. (1997) Role of environmental contamination in the transmission of vancomycin‐resistant enterococci. Infect Control Hosp Epidemiol 18: 306–309. 9154471 10.1086/647616

[mbt212755-bib-0040] Weber, D.J. , Rutala, W.A. , Miller, M.B. , Huslage, K. , and Sickbert‐Bennett, E. (2010) Role of hospital surfaces in the transmission of emerging health care‐associated pathogens: norovirus, *Clostridium difficile*, and Acinetobacter species. Am J Infect Control 38: S25–S33. 20569853 10.1016/j.ajic.2010.04.196

[mbt212755-bib-0041] WHO, F. (2001) Health and Nutritional Properties of Probiotics in Food Including Powder Milk with Live Lactic Acid Bacteria. http://www.fao.org/3/a-a0512e.pdf: World Health Organization [online].

